# Older Adults’ Perspectives and Experiences With Digital Health in Singapore: Qualitative Study

**DOI:** 10.2196/58641

**Published:** 2024-11-11

**Authors:** Qiao Ying Leong, V Vien Lee, Wei Ying Ng, Smrithi Vijayakumar, Ni Yin Lau, Ingela Mauritzon, Agata Blasiak, Dean Ho

**Affiliations:** 1 The Institute for Digital Medicine (WisDM), Yong Loo Lin School of Medicine, National University of Singapore Singapore Singapore; 2 The N.1 Institute for Health, National University of Singapore Singapore Singapore; 3 IVV Labs AB Lund Sweden; 4 Department of Biomedical Engineering, National University of Singapore Singapore Singapore; 5 Department of Pharmacology, Yong Loo Lin School of Medicine, National University of Singapore Singapore Singapore; 6 The Bia-Echo Asia Centre for Reproductive Longevity and Equality, Yong Loo Lin School of Medicine, National University of Singapore Singapore Singapore

**Keywords:** digital health, gerontology, geriatrics, elder, aging, Singapore, qualitative, mHealth, mobile health, experience, technology use, interview, perspective, acceptance, technology adoption

## Abstract

**Background:**

Technology use among older adults is increasingly common. Even though there is potential in leveraging technology to help them manage their health, only a small fraction of them use it for health-related purposes.

**Objective:**

This study seeks to understand the perspectives of and experiences with digital health (DH) among older adults in Singapore.

**Methods:**

A total of 16 participants (age range 60-80 years; n=11, 69% female) were interviewed for approximately an hour (range 27-64 minutes) about their health, DH use, and DH experiences. The interviews were recorded, transcribed verbatim, and thematically analyzed.

**Results:**

Five main themes emerged from the interview: support in developing DH literacy, credibility, cost and benefit considerations, intrinsic drive to be healthy, and telehealth. Older adults need support in familiarizing themselves with DH. When considering DH options, older adults often relied on credible sources and preferred DH to be free. Monetary incentives were brought up as motivators. The intrinsic drive to live longer and healthily was expressed to be a huge encouragement to use DH to help obtain health-related knowledge and achieve healthy living goals. The idea of telehealth was also appealing among older adults but was seen to be more suited for individuals who have issues accessing a physical clinic.

**Conclusions:**

Our findings offer insights into the various aspects that matter to older adults in the adoption of DH, which in turn can help reshape their health-seeking behavior and lifestyle. As such, policy makers and DH implementors are encouraged to take these into consideration and align their strategies accordingly.

## Introduction

The global population of older adults aged 65 years and older is expected to increase in the coming years. In 2015, there was an estimated 8.5% of individuals aged 65 years and older globally, and this proportion has been projected to grow to 16.7% by 2050 [1]. In Singapore, the rapid increase of an aging population is evident given the growth of the proportion of older residents from 11.7% in 2013 to 19.1% in 2023 [2]. As the median age of Singapore continues to increase (median age is 43 years in 2023), it is projected that 24.1% of the Singaporean population will be aged 65 years and older by 2030 [2]. While increased longevity can be attributed to the success of medical advancements and improvements in public health practices [3], longevity is often accompanied by an increased risk of chronic diseases and a decrease in physical and cognitive function [4]. Beyond disease burden, multiple chronic diseases in older adults have been associated with greater health care use and costs [5]. Thus, it further contributes to the increasing load on the health care system and patients themselves, which is evident in Singapore as the acute hospital admissions [6] and duration for hospitalization episodes [7] were both reported to be rising.

To meet the health demands of a growing aging population presented within Singapore, the application of digital health (DH) to encourage these healthy behaviors serves as a valuable tool to assist in the journey toward a healthy aging population. DH for an aging population can include wearable devices for health monitoring, telemedicine, and mobile phone apps [8]. While the potential of DH for health behavioral change is still being tested, findings to date have shown promising results in promoting health behaviors and improving health-related outcomes [9-11]. Such an approach can serve as an invaluable tool to assist older adults in their journey toward healthy aging through preventive health, active aging programs, and care services. Accordingly, Singapore’s 2023 action plan for successful aging aims to empower seniors to take charge of their physical and mental well-being through the engagement of regular group exercises, adopt a healthy and balanced diet, and go for regular health screenings and follow-ups [12]. Nevertheless, the success of a digital tool is dependent on its uptake and sustained use by the intended population. Despite a high proportion of older adults in Singapore owning at least 1 digital device (38.1% have 1 digital device and 55.82% have 2 or more digital devices), only a small number of older adults use their devices for functional purposes such as telehealth services (10.5%) [13].

Expanding the use of DH to older adults will benefit from significant input from older adults themselves to ensure that the tool is designed around their needs, capabilities, and preferences [8]. Through engaging older adults during the design process, DH innovators can better understand what drives DH use, which can then inform downstream design considerations and implementation strategies when rolling out the tool. While Singaporean older adults consider DH useful in health management, they do not consider health care–related technology critical for their daily lives [14]. Furthermore, there is generally a strong preference for direct contact with health care professionals among this group [15].

This study aimed to explore Singaporean older adults' perspectives of DH and their experiences with existing DH tools. Through this, the study sought to understand what matters to Singaporean older adults when using DH to encourage improved health behaviors.

## Methods

### Recruitment

We recruited individuals through purposive sampling from the community in Singapore through outreach sessions and social media platforms (ie, Telegram and Facebook). Interested participants responded to advertisements by reaching out to the study team via email or text message, and they were provided with the participant information sheet and informed consent form. Participants were screened based on the following inclusion criteria: (1) age of 60 years and older and (2) have experience using health-related technologies. Recruitment took place over a period of 1 year (November 2021 to October 2022). None of the participants refused to participate or dropped out after consenting to the study.

### Data Collection

This study used semistructured interviews to understand the older adults’ perspectives of and experiences with DH in Singapore. Each interview was approximately 60 minutes and conducted either in person or via Zoom depending on the participants’ preference. In-person interviews were conducted in a quiet public space (eg, conference room, cafes, and void decks). All interviews were conducted in English with at least 2 researchers from the data collection team—QYL, VVL, SV, WYN, and NYL—present. The all-female data collection team consists of 2 postdoctoral fellows and 3 research assistants (with bachelor’s degrees). All researchers who collected interviews had prior training in qualitative data collection and analyses. To minimize interview bias, a standardized interview protocol and guide were used (Multimedia Appendix 1). All researchers in the data collection team underwent pilot sessions before the interviews. Peer feedback was provided during the pilot sessions. Relationships with the participants prior to the interviews were established through email, call, or messages. Participants were told about the researchers’ involvement in the study and the reasons for doing the research. At the beginning of the session, informed consent was obtained, and all participants consented to audio recording of the interview for transcription purposes. Open-ended questions were asked during the interview to understand the participants’ health care journey, their perspectives, and their experiences with DH. This paper focuses on the DH aspect of the interviews. At the end of the session, all participants were reimbursed for their time. All data collected, including signed consent forms and interview recordings, were deidentified, encrypted, and stored in a secure database. No repeat interviews were conducted, and transcripts were not returned to participants for comments.

Data collection ended when data saturation was achieved, and the sample had reached maximum variation by age, gender, and ethnicity.

### Data Analysis

All of the interview recordings were transcribed verbatim and thematically analyzed using the method described by Braun and Clarke [16]. Each transcript was randomly assigned to a member of the data collection team for inductive coding. All coders familiarized themselves with all interview transcripts and initial coding was conducted through a process of descriptively labeling segments of data. Each coder independently analyzed their assigned transcript. As the coding process progressed, the data collection team consistently reviewed and agreed on the coded data collectively and subsequently resolved any discrepancies through discussion. Categorization of the codes was conducted by QYL, who grouped the labeled data into categories and subsequently into broader themes. Data saturation was achieved when no new theme was identified. Grouping of themes was tracked on a master Excel workbook, and data saturation was confirmed through discussion with all researchers who were involved in data collection and analysis. The final codes and themes were based on the discussions and iterations with VVL, and a final review with the other members of the data collection team. The transcribing and analyzing steps were conducted using Microsoft Word and Excel respectively [17].

To ensure credibility and confirmability, reflexivity was applied throughout the data collection process. Team discussions were conducted at the end of each week. During team discussions, the researcher’s interaction with participants and thoughts on the interview was discussed and noted down. Furthermore, peer debriefing was conducted throughout the interview and coding process to obtain feedback from other members of the data collection team and minimize personal biases. As the study team had specific interests in DH, it may have influenced the team to engage with participants about DH in a positive manner. During each discussion, the study team made note of the positives and negatives in relation to DH and were consistently reminded to prompt participants about both sides. To ensure transferability and dependability, sampling strategies, research procedures, and thick descriptions are provided in the manuscript.

### Ethical Considerations

The National University of Singapore Institutional Review Board approved this study (NUS-IRB reference: NUS-IRB-2021-12). All participants provided informed consent and were informed of their right to withdraw from the study at any time. All data collected from participants were de-identified. Participants were reimbursed SGD 30 (US $22.60) for their time and participation.

## Results

### Participant Demographics

The study sample consisted of 16 participants, with an age range of 60 to 80 (mean age 66.9, SD 5.9) years. They were of Chinese (9/16, 56%), Indian (5/16, 31%), and Malay (2/16, 13%) descent and were fluent in English. A total of 11 (69%) participants were female. Hypertension, hyperlipidemia, diabetes, and cardiovascular diseases were some common medical conditions reported by the participants. Other medical conditions brought up less frequently included history of breast cancer, psychosis, ulcerative colitis, and shingles. Participant demographic data are presented in Multimedia Appendix 2.

During the interview, participants described their experiences with a variety of technologies. These technologies, consisting of mobile apps, web-based platforms, and DH devices, were used for multiple purposes as listed in Table 1.

**Table 1:** Technology use of participants.

Category	Technologies used
General	Zoom, Google, YouTube, Lazada App, Singtel App, and Amazon Alexa
Social media	Facebook, WhatsApp, and Telegram
Health-specific mobile apps and webpages	HealthXchange.sg, Healthy365 App, ActiveSG App, SAFRA Mobile App, HealthHub App, NUHS App, and Mayo Clinic
Health-specific devices	Bluetooth-connected Glycoleap App, Abbott Freestyle Glucose Monitoring device, FitBit, blood pressure monitoring device, and oximeter

### Interview Data

Five themes emerged from the interview data. The themes consist of support in developing DH literacy, credibility, cost and benefit considerations, intrinsic drive to be healthy, and telehealth.

### Support in Developing DH Literacy

#### Overview

The first theme—support in developing DH literacy—refers to the endeavor to improve DH literacy among older adults. This support builds upon the foundation laid by the existing awareness of DH through education and accessibility. The participants often learned about the existence of DH by word of mouth through family, friends, and health care professionals. Another way in which DH was made aware among older adults was through promotional materials disseminated through mass media namely newspapers and social media platforms (eg, Facebook).

#### Education

At the initial stage of DH adoption, education was crucial to ensure participants familiarized themselves with the DH interface, functionality, and navigation. Besides familiarization, education provided the support needed to sustain DH use. The overview of how the older adults might go from not knowing to knowing was described by participant 15, “Now the old people also trying to catch up, but someone [has] to educate them right...how to use it. Nowadays, there are the workshop[s] for elderly, how to use the app[s], how to use Tiktok, Facebook and all that. So, it [will] take some time to...give them teaching, [it] will take time [for them] to learn.”

When asked about thoughts on creating awareness and bridging that to actual DH use through education, participant 5 described, “I think it involves...some outreach, educational programs, so people will come to know.” The participants added that outreach sessions, education programs, and workshops at community centers and hospitals are good starting points. Participant 16 shared that community centers were an ideal point of contact “because they come to the community centre to spend time sometime[s]” but also specified that this option was only available for “those who can walk.” Upon further elaboration, the same participant also shared, “The best place [to spread DH awareness] is the hospital. Hospital is the only place you can get that in...The nurses can educate them that these avenues are also open.”

These sessions should offer guidance and troubleshooting support through step-by-step instructions and video tutorials. Each session should preferably be one-to-one and in person “to get seniors used to the concept...[and] to explain to them...it’s non-threatening, it’s helpful,” as shared by participant 11. With repeated familiarization over time and easier access to avenues for education, older adults could become more receptive to DH. However, this process can be very time-consuming as it can be repetitive in nature and would require immense patience and time.

#### Accessibility

Accessibility reflects the ability of older adults to reach and use DH. Even though there was an intrinsic drive to learn and explore DH platforms available, participants expressed discomfort over unfamiliarity and poorer cognitive abilities. Participant 10 described, “No chance to learn [about DH]. If there is opportunity, then we pick here and there. So, sometimes we are struggling with it...The young ones will be very good. You need to start playing since young. For us...[we are] a bit slower, memory also weaker. So, what we have done earlier, we may not be able to remember. So, it’s very difficult to do it on your own to try to learn.” Concerns about slower cognition and poorer memory led to lower confidence in their ability to use DH, thereby creating barriers to DH adoption.

Support and guidance from “someone at home or...some neighbours” could act as a push factor “to help them [the older adults] understand certain things [DH platforms]” to overcome the aforementioned barriers to DH adoption, as shared by participant 11. Support beyond their own family members was especially important as pointed out by participant 6, as “the singles [elderly]...have no one to help them, they can go to the digital ambassadors.” These digital ambassadors could potentially be the younger generation. To illustrate this point, participant 11 shared, “I got [tech] problem here. How? Anybody know? Things like that. And if you have a handphone, you look for a younger colleague...So, sometimes you can [ask them], ‘help me’.”

### Credibility of DH Platforms

#### What and Who Do They Trust

Trust emerged to be essential in the health management of older adults. Although the information was described to be readily available, the participants expressed the need to sieve through it and had to rely on trusted sources to assist in their decision-making regarding their own health. Rather than having just 1 trusted individual or organization, it was common for older adults to seek health-related information or DH platform recommendations from multiple sources such as social networks or health care professionals. For instance, participant 1 expressed, “I always trust my friend. What they tell me to do, usually I will do it” while adding on, “If doctor say that I need this, of course I will trust the doctor”. The mention of recommendations from friends emerged repeatedly. This was attributed to the trust in their friends’ testimonials to ascertain the reliability of DH platforms. In participant 15’s case, concern from friends encouraged them to take their health seriously and that included adopting DH to manage their health. “I just wanted to try out...They are so concerned [of me], they say, you are putting on weight...you must check up your health...So, your friends are so concerned about you...why not I take control for myself, that’s why I take it seriously.”

While information or suggestions from doctors or health care institutions such as hospitals and clinics were perceived as trusted sources, other individuals or organizations that made up the trusted circle of the older adults also included family members, friends, neighbors, governmental organizations (eg, Ministry of Health) and higher learning or research institutions (eg, National University of Singapore).

#### What Matters in Establishing the Trust

The DH recommendations from health care professionals were built on the existing doctor-patient relationship. Several considerations were brought up as crucial in forging and strengthening that bond. For instance, participant 7 expressed, “I think if the doctor has been with me for quite a while and I trust the doctor, it should not be an issue”. Beyond that, the type of artificial intelligence used in the DH, the length of medical practice as well as the level of expertise (eg, junior doctors and consultants) of doctors recommending the DH also mattered while forging this sense of trust. Participant 7 further expanded, “It depends on the doctor himself, the level of expertise. I mean you can’t compare between a houseman or a student doctor, to a consultant, right? So, to have my level of assurance that I can trust them fully, I think it depends on the level of expertise and the experience—the experience of the doctor.”

Apart from the source of DH recommendations, the mode of information delivery was also crucial. For example, vital pieces of information to establish trust could be delivered in the form of statistics. Participant 14 described a possible way of conveying this information, “Based on your precedence [medical history] and all that kind of thing, [the DH] will tell them [the users], how many percent has actually...done this and they have recovered...Some stats based on the recovery rate, that kind of thing...I think that helps.”

Furthermore, there were other considerations that cemented this trust. For example, participant 9 mentioned, “I’ll ask him [the doctor], is there any risk [of] like you go on an app and do it yourself? That way I don’t mind”. The participants also emphasized the need for DH platforms to be validated and substantiated by evidence in order to be deemed as trustworthy tools in health management.

#### What Makes Them Lose Trust

“Having to see the doctor personally, and he diagnose...and do it for us, that’s all I want. I would rather do that,” said participant 9, who felt that DH platforms were not as trustworthy as doctors due to the lack of human touch. Cybersecurity concerns also emerged as a factor that would erode trust in DH use. For instance, participant 13 expressed, “I don’t find [it] comfortable...when you go to the app, [and] they ask for Singpass...sometime, you may click the wrong button or you go to URL, they might take...all your information...then from there phish.”

### Cost and Benefit Considerations

#### Types of Incentives

Two types of incentives were described by the older adults as follows: monetary incentives and results from DH engagement.

“Number one of course monetary...Even if you give me an NTUC [a local supermarket chain] voucher, it is still money”, said participant 10. The participants described that the use of gamification and financial incentives while using the DH platforms had been an effective and creative way for them to engage in DH use. The distribution of free DH devices, such as wearables, was also quoted to have similar motivating effects as financial incentives. For example, ActiveSG (Singapore government health promotion program) has 100 Singapore dollars preloaded to incentivize the onboarding of potential users and promote sustained use. As described by participant 6, “If you don’t learn...it’s forfeited from you. Then you are forced to learn.”

Without incentives, the participants were more inclined to use DH platforms simply as a means of tracking health metrics. After the initial engagement, observable health outcomes from their DH use seemed to be the driver for sustained use. To illustrate this point, participant 12 described, “I have to see the result...maybe after a month or two. If they say do this, do that, you will lose your weight, I have to see whether it happens. After that, yes, I’ll use. But I don’t mind taking the first step, yes. But after that time, if I feel it’s of no use, I will stop it.”

#### What Incentives Enable Them to Do

Two health-seeking behaviors consistently emerged in the data when incentives were involved—staying active and opting for healthier dietary options.

Regarding the use of incentives to encourage users to stay active, participant 6 mentioned, “They put some codes around different places for you to go and find and scan...If I am passing by [and] I know that there is a place to scan to get points, then I just make a detour...So, they got all these challenges inside [the app] to keep you more active and maybe more mentally alert.”

Furthermore, the participants were highly motivated to opt for healthier food options when incentivized by a point system through a DH platform. For example, participant 7 expressed, “That’s how I accumulated [points], this QR code from buying food...with the healthier choice logo.”

#### Cost

Generally, participants preferred DH use to be free. Participant 6 shared, “[The DH] must be available free, if you ask people to pay, they will be put off” and further elaborated, “I go for all the free things. Because this cost, sometimes I don’t feel is justified.” While there was a clear preference for free DH platforms, some participants expressed that they did not mind paying a one-off or subscription fee, but only if the cost was reasonable and the DH proved to be beneficial.

### Intrinsic Drive to be Healthy

#### Overview

“Health is already a motivating factor, nobody wants to be unhealthy.” Participant 6 succinctly summarized that this intrinsic drive, along with the aspiration to live longer and healthier, motivated older adults to adopt health-seeking behaviors. DH platforms were described to have the potential to help participants in their pursuit of realizing their healthy living goals and gaining knowledge out of necessity, or curiosity.

#### Healthy Living Goals

Participant 11 emphasized that addressing health-related concerns should be approached holistically - catering to both physical and mental well-being. While concomitant conditions were common, participant 7 shared, “You must treat the patient as a whole, not compartmentalize them into health issues like mental, diabetes, heart.” The idea of personalized health-related tracking also appealed to older adults. For instance, participant 10 shared that diabetic patients were motivated to use glucometers to understand how different food choices affected their blood sugar levels. Furthermore, the relevance of the materials offered by DH platforms heavily dictated their use among older adults. Although the idea of relevance varied according to each older adult’s area of interest (eg, diet, cultural practices, sleep, and medical concerns), there was a consensus that DH platforms available were too based on Western culture, too general, and lacked the touch of personalization. When seeking knowledge-based content, participant 8 shared, “ [The DH should be] a one-to-one consultation and give you information that is relevant to you, specific to you, not so general that I might as well go Google you know”. This highlighted the preference for more personalized content over generic information. Another example of relevance was based on the idea of localization. Participant 14 described that “a lot of [DH platforms] are...catered to Western kind of...audience.” Accordingly, they may not be relatable to the Asian context and users. These examples demonstrate that irrelevance can impact the perceived relatability of DH platforms.

The participants expressed various aspects where DH can assist in health management and attainment of healthy living goals. First, DH platforms should have provided feedback especially if gamification was incorporated. Such feedback could be a report of their performances based on a benchmark, or actionable recommendations based on individual health concerns. Second, participant 16 suggested that DH platforms could serve as the “one tool for common disease[s] like diabetes or high blood pressure.” Third, nudging as a form of reminder, as described by participant 15, could be “good, very helpful, like someone taking care and telling me...you must take this, you must do this.”

#### Knowledge

While achieving one’s health-related goals is important, participants also expressed that this pursuit needed to be supplemented by knowledge. Most described using DH out of necessity or curiosity. Participant 11 expressed, “I think that the onus is on me to do my own reading and find things also, to be more knowledgeable about my own issues.” This motivation for knowledge was not only described to be driven by the goodness of oneself, but it also extended to their partners. Such motivation was described by participant 12 who mentioned, “My husband is diabetic, so I try to look for items which is better for him”.

As such, the older adults generally appreciated health information and recommendations deployed through DH as supportive of their aspirations to live healthily. For example, participant 7 shared, “The [web-based] articles from SingHealth (health system in Singapore), when I got my treatment for my oral health...so they started giving me all these articles every now and then which I would read up.” Participant 8 also described, “Now, [the] internet [is] so convenient. A lot of YouTube [videos] advise how to...take care of diabetes...fasting...that kind of thing. So, I try everything.”

Beyond knowledge-based information, DH was also described as an ideal avenue to broadcast events that support healthy and active living. For example, participant 15 shared that happenings of interest including “a weekend activity...group walking activity, or running, simple exercise, Tai Chi...all kinds of simple exercises” could be broadcasted through DH, which could subsequently enable “most [in] my age group [to] get together and then become more active, then you get to know people”. A similar sentiment was expressed by participant 1, “I will use that [DH] if they got social [events], they will tell me, where can [I] go.”

Furthermore, the accessibility of web-based information also enabled health-seeking behaviors, such as in the case of participant 15 who shared, “During the COVID time, I was afraid to take the COVID injection, so I read through more [on websites], now I’m happy [to take the injection].”

### Convenience of Telehealth

The appeal of telehealth is centered around the convenience that it offers. Telehealth not only allowed the participants to seek medical attention remotely, but it also eliminated the need and hassle of traveling to a clinic or health care institution. Participant 5 shared that their interaction with the polyclinic through telehealth made him “feel better because [there is] no need to walk all the way there [the polyclinic].”

Furthermore, the prompt responses by the medical team also made telehealth an ideal feature in DH. Participant 15 described, “It’s a good idea that nowadays...to interact with the phone, and anything you can talk faster. I think during the COVID time, it started very well...I got COVID also, so they consult me to tell me [via] this thing [telehealth], then advise me the medication, they send me through phone, then they deliver to my house. It’s quite [a] good idea.” This also demonstrates how the recent COVID-19 pandemic accelerated the shift in favor of telehealth adoption.

Nevertheless, despite the boons that telehealth offers, the participants generally still preferred to consult the doctors in person. However, they also acknowledged that under some circumstances, telehealth might be more ideal. For example, participant 1 shared, “Last time is okay...[I] don’t mind going out. But nowadays, [I am] usually very scared to go out, especially for the elderly because we are more prone to the disease. So, [it] is good that...I make appointment and zoom doctor. [The doctor] tell us, your results...you must take care, then is good. Better than we travel there for the results.” Other groups of individuals who were identified to potentially benefit from telehealth included those who “are really very sick...and are not able to move”, as shared by participant 12.

## Discussion

While it is usual for older adults to use technology, using DH is not as common [18]. With the added challenges stemming from the natural progression of aging—slower cognition [19,20] and poorer memory [21]—overcoming the learning curve of DH can be daunting. Awareness and accessibility form the foundation to overcome the learning curve of DH, which in and of itself is a multi-step process. The building blocks leading to DH adoption and subsequently sustained use can be facilitated through various factors, some of which are unique to the Singaporean context, and will be discussed in this section.

While awareness of DH can be achieved through DH exposure, it is not always sufficient for obtaining access to DH. Accessibility issues expressed by the participants in this study generally centered around the need for support and assistance to help them to familiarize themselves and use the different aspects of DH (eg, interface, navigation, and functionality). Whether it was contextualized learning materials targeted to the masses or personalized guidance by DH ambassadors, participants described education as a necessity to overcome the steep learning curve—a barrier for DH uptake. Similar findings are echoed in a review that reported difficulty in technology use as the most common patient-level barrier to DH technology uptake, while facilitators included empowerment, education, and training sessions [22]. Overcoming this barrier is a mammoth effort, which was also echoed by the older adults in this study since it would require significant resources (ie, manpower, time, and funding) to ensure continual DH support ranging from step-by-step guidance to troubleshooting expertise. This understanding from our study, along with the existing qualitative work that provides invaluable insights regarding the general perception [14,22,23] and the user preferences [24] of DH among the older adults in Singapore, are critical in informing policymakers and DH implementers, and in aligning strategies that promote DH adoption for its potential to be realized and benefits reaped.

Trust repeatedly emerges as an important factor in technology adoption, including in other populations such as women [25,26], Korean Americans [27], and patients with cancer [28]. Like the findings in this study, individuals in those studies tended to seek health-related information from multiple sources—often from health care professionals, family, and friends [26,29], which were reported to be credible. Different sources of information are used to compare, evaluate, and triangulate the information as a means of filtering the vast body of data available. The older adults in this study expressed trust in physicians and preferred to cross-check health information with them. Interestingly, even though the older adults expressed trust in physicians, several nuances mattered in establishing this trust. For instance, the older adults in this study expressed that if a physician was recommending a novel technology, they were more comfortable following the recommendation if the physician had vast experience in the technology and was a seasoned medical practitioner (eg consultant over a junior doctor). Such nuanced behavior associated with trust was also reported in a study involving Singaporean older women [29], where information regarding health-related products was not taken at face value but was closely judged. Furthermore, it is worthwhile considering the influences of ethnic norms, linguistic capabilities, and level of education in instilling trust in DH among older adults in a culturally and racially diverse country like Singapore. In 2 studies—1 in Singapore [29] and 1 in the United States [27]—ethnic language, dialects, and linguistic capabilities might have played a role in establishing trust. Additionally, it is worthwhile noting that Singaporeans are generally trusting and responsive towards most government-related directives. This is especially evident in the COVID-19 vaccination uptake during the recent pandemic. Vaccination status was shown to be positively predicted by trust in formal sources (ie, government services, local television, and radio), but not by trust in informal sources (ie, family and friends) [30].

Older adults in this study emphasized the importance of having both extrinsic and intrinsic motivation to drive DH adoption and sustained use. Extrinsically, older adults mentioned financial incentives and tangible feedback about the results from their behavior change as drivers that would encourage greater DH use. This is in line with one of Singapore’s nationwide mobile health physical activity programs, The National Steps Challenge deployed via the Healthy365 (Health Promotion Board) app, which was able to successfully reach about 26% of the Singapore population (ie, 1.3 million out of 5.0 million as of 2019) [31]. Key factors that were cited to contribute towards the success of the program’s reach include monetary reward in exchange for Healthpoints (collected through steps) and free fitness wristbands offered to participants [31]. Beyond the need for external motivators, older adults in this study also demonstrated an intrinsic drive to be healthy and highlighted the potential of DH in aiding them to achieve health-related goals through education and actionable recommendations. This need for both intrinsic and extrinsic motivation in DH tools targeted towards older adults was also described in a recent qualitative systematic review, which emphasized the need for users to first be motivated to make a change in their life with the use of extrinsic motivation to aid in device adoption and value adding towards the users’ life [32]. Accordingly, DH that is being developed to induce healthy behavior change in older adults should consider tapping into both types of motivation to strengthen adoption and engagement rates.

When asked about telehealth, older adults in this study were generally receptive and brought up convenience as its main benefit. While the COVID-19 pandemic may have facilitated the shift in perception in favor of telehealth in the eyes of older adults, participants in this study still preferred in-person consultation. The preference for in-person consultation was also reported in another Singaporean qualitative study exploring the attitudes of older Singaporeans towards telehealth [33]. Furthermore, the overall awareness among Singaporean older adults about the range of telehealth services available remains low with more than 50% of participants not knowing about telehealth [33]. A consideration suggested by older adults in this study was to focus telehealth efforts on individuals who would benefit from telehealth the most (eg, immunocompromised individuals and individuals with mobility concerns). Targeted strategies to reach older adults who face barriers to in-person consultations could ensure that the technology is being used by groups of older adults whose health care experience may be enhanced the most through the adoption of telehealth.

Due to the linguistic constraints of the data collection team, the study only recruited older adults who were English speakers. As such, older adults whose first language was not English (eg Mandarin, Malay, Tamil, Hokkien, and Cantonese) were excluded. Accordingly, the study may not capture nuances that may be specific to non-English speakers. Furthermore, the recruitment strategies of using social media platforms and community outreach might miss segments of the population not active in these areas. For instance, older adults with lower socioeconomic status or socially isolated may not be captured through these recruitment strategies. Nevertheless, this study was able to explore the perspectives of older adults from the 3 major ethnicities in Singapore (Chinese, Indian, and Malay) with varying levels of health conditions. As the aim and the design of this study were to understand the perspectives of older adults in Singapore through a qualitative study, the findings contribute to the knowledge about DH in the Singapore context. To further strengthen the findings from this study, next steps can include the use of data triangulation to corroborate current findings using a different data source (eg, focus groups or observational data), and increasing the sample size or interview length to enhance comparability and generalizability of the study findings. Moreover, strategies toward the development and implementation of DH should be context-specific, and an application of the findings outside of Singapore may require additional validation.

The process of aging creates an inevitable burden not only on an individual but also on the health care system and society. A potential way to help ease this burden is to harness the potential of technology, specifically through DH. This study demonstrated the various perspectives of and experiences with DH among older adults in Singapore. DH developers and implementors are encouraged to take these into consideration to improve DH adoption and implementation within this population, and to align their strategies accordingly.

## Appendix

Multimedia Appendix 1 Study recruitment and interview protocol.

Multimedia Appendix 2 Demographic data of participants.

## Abbreviations

DH: digital health
