# Supplementary material for: Older Adults’ Perspectives and Experiences With Digital Health in Singapore: Qualitative Study
Source: JMIR Hum Factors. 2024 Nov 11;11:e58641. doi: 10.2196/58641 (PMC11589501; doi:10.2196/58641)
Supplement: Multimedia Appendix 1 [file humanfactors_v11i1e58641_app1.docx]

**Multimedia Appendix 1**

**Screening**

Interested participants were contacted and briefed about the study via email, text or phone call. During the initial engagement, the research team obtained information about their age, gender, medical condition and digital health usage. Additionally, a digital copy of the Participant Information Sheet and Informed Consent form was sent via email or text to the participants for further reference.

**Organising interview**

Participants who would like to proceed with the interview would be followed up via email, text or phone call, where details regarding preferred day, time and online or in-person were obtained and confirmed. Subsequently, the interview details were sent out after scheduling took place and the day before the interview.

**Pre-interview**

Prior to starting the interview, researchers introduced themselves and their role in the project and institution. The research team reintroduced the aim of the study and reiterated the study procedure with the participants. In-person participants were briefed using the physical copy of the Participant Information Sheet & Consent Form while online participants were briefed using the share screen function on Zoom. At the end of the briefing, participants were asked if they had any questions regarding the study. When the participants were happy to proceed with the study, the participants were requested to sign the Consent Form. Online participants were given control of the screen and asked to sign via Zoom.

Following the signing of the consent form, participants were asked if they were comfortable to begin the interview. If so, researchers began recording using two different audio recorders and began the interview. The interview guide below was utilised during the interview.

**Interview guide**

1. **Healthcare journey**

- Could you tell me about your journey with healthcare?
- What types of medical treatment are you doing?
- How do the medical treatments that you are doing make you feel?
- Tell me about what happens when a new treatment is introduced. What information do you get? What happens next?
- What makes you feel like you would like to try a new therapy?
- When do you feel like you would like to skip it?
- Can you tell me about a situation when you skipped treatment?
- Could you describe a treatment that you wanted to do?

1. **Experience with and perspective of digital health**

- Do you use technology? Can you describe your usage?
- What is easy/difficult about technology usage? Can you provide examples?
- Have you come across/are using any health-related apps? Do you use technology for any health-related reason?
- Can digital health improve your everyday life in any way? Please describe.
- What is your idea of digital support for your health? What kind of technology-based support would you like to see?
- Can digital health present any difficulties for you? Please describe.
- What are some preferred modes and features you would like to see?
- Do you think there can be problems in using technology for health?
- How can these problems be overcome?
- What might help you use technology more generally and for your health?
- What should an incentive to help you use technology look like?
- What might stop you from using technology generally and for your health?
- What might make you discontinue using any technology generally and for your health?

**Post-interview**

Before the end of the interview, participants were asked if there was anything else they’d like to contribute before pausing the audio recording. When participants were satisfied with what they have shared, the study team stopped the audio recording.

The study team thanked the participants for their time and proceeded to reimburse the participants with SGD30. Participants were reimbursed using cash or PayNow (a digital service offered by Singapore banks to send and receive money using the bank’s phone application). Participants were required to sign a receipt to acknowledge the reimbursement. Participants were told to recontact the study team if they had any questions regarding the study or changed their mind about any information shared during the interview.
