# Supplementary material for: Older Adults’ Perspectives and Experiences With Digital Health in Singapore: Qualitative Study
Source: JMIR Hum Factors. 2024 Nov 11;11:e58641. doi: 10.2196/58641 (PMC11589501; doi:10.2196/58641)
Supplement: Multimedia Appendix 2 [file humanfactors_v11i1e58641_app2.docx]

**Multimedia Appendix 2.** Demographic data of participants

| Participant | Age | Gender | Ethnicity | Medical conditions |
| --- | --- | --- | --- | --- |
| 1 | 69 | Female | Chinese | None |
| 2 | 63 | Male | Chinese | Diabetes, high blood pressure, high cholesterol, CVD |
| 3 | 72 | Female | Chinese | Macular hole |
| 4 | 80 | Female | Chinese | History of breast cancer, CVD |
| 5 | 62 | Female | Chinese | Hypertension, hypothyroid, high cholesterol |
| 6 | 64 | Male | Indian | Ulcerative colitis |
| 7 | 60 | Female | Malay | Psychosis |
| 8 | 62 | Male | Chinese | Diabetes |
| 9 | 65 | Female | Indian | None |
| 10 | 66 | Male | Chinese | Diabetes, high blood pressure, high cholesterol |
| 11 | 72 | Female | Chinese | Rheumatoid arthritis, high cholesterol |
| 12 | 71 | Female | Indian | None |
| 13 | 76 | Female | Chinese | Osteoarthritis, foot corn |
| 14 | 63 | Female | Malay | History of breast cancer |
| 15 | 60 | Male | Indian | Diabetes, high blood pressure, CVD |
| 16 | 65 | Female | Indian | Diabetes, high blood pressure, CVD, respiratory condition, vision issues, shingles |

Note: CVD, cardiovascular disease.
